# The impact of sputum sample quality on sputum culture results: a retrospective analysis of clinical microbiology sputum culture data spanning 10 years

**DOI:** 10.3389/fmicb.2026.1894814

**Published:** 2026-07-07

**Authors:** Kunzhan Dong, Hong Yan, Jie Zheng, Jun Luo, Jia Li, Zhifeng Zhang, Shicong Wang, Shuo Gao, Chang Liu

**Affiliations:** 1 Department of Clinical Laboratory Medicine, Nanjing Drum Tower Hospital, Affiliated Hospital of Medical School, Nanjing University, Nanjing, China; 2 Laboratory Medicine Center, The Second Affiliated Hospital, Nanjing Medical University, Nanjing, China; 3 Department of Laboratory Medicine, The Fifth Affiliated Hospital of Anhui Medical University, Anhui, China

**Keywords:** antimicrobial resistance, BALF, diagnostic sensitivity, lower respiratory tract infection, sputum specimen quality

## Abstract

**Background:**

Despite advances in molecular diagnostics, sputum culture remains fundamental for the microbiological diagnosis of lower respiratory tract infections. Current guidelines recommend rejecting unacceptable sputum specimens, yet their actual diagnostic yield, pathogen distribution, and impact on antimicrobial resistance assessment have not been systematically evaluated using large-scale data. A retrospective analysis was performed on 143,101 sputum culture and quality assessment records collected at Nanjing Drum Tower Hospital between 2015 and 2025. Specimens were classified as acceptable or unacceptable according to the Murray-Washington criteria, with the same period bronchoalveolar lavage fluid cultures included as comparator group.

**Results:**

The overall culture-positivity rate was significantly higher in acceptable than in unacceptable specimens (57.28% vs. 50.87%; Odds Ratio = 1.29, 95% CI: 1.27–1.32, *P* < 0.001). Dominant organisms, including *Klebsiella pneumoniae* and *Acinetobacter baumannii*, were detected at higher rates in acceptable specimens, whereas yeast-like fungi were significantly enriched in unacceptable sputum, indicating oropharyngeal contamination. Filamentous fungi (e.g., *Aspergillus* spp.) showed a stepwise decreasing trend in BALF > acceptable sputum > unacceptable sputum. Antimicrobial resistance rates of *K. pneumoniae*, *Pseudomonas aeruginosa*, and *Staphylococcus aureus* were significantly higher in sputum isolates than in BALF isolates, suggesting that sputum cultures may overestimate resistance risk.

**Conclusion:**

The large-scale findings support cautious, contextual interpretation of unacceptable sputum results, particularly when better specimens cannot be obtained. This approach helps preserve critical diagnostic information and avoid unnecessary antimicrobial escalation.

## Introduction

Despite the widespread clinical application of molecular biology detection techniques in recent years, traditional bacterial culture remains the most fundamental and commonly used laboratory method for the microbiological diagnosis of respiratory infections (Joyce, 1986). Among various respiratory specimens, sputum is the most common and relied-upon specimen type in clinical practice due to its non-invasiveness and ease of acquisition (Saukkoriipi et al., 2019). Ideally, sputum can directly reflect the infection status of the lower respiratory tract (bronchioles, alveoli); however, during patient self-collection, some sputum samples inevitably become contaminated by the normal colonizing flora of the upper respiratory tract and oropharynx, producing “diagnostic noise” that interferes with clinical judgment (Peng et al., 2020). To mitigate this risk, current guidelines and standards strictly require laboratory personnel to perform microscopic evaluation of sputum sample quality before culture, retaining only acceptable samples for subsequent processing to ensure the reliability of test results (Miller et al., 2024). The guidelines emphasize that specimen quality itself is not inherently problematic, but in real-world clinical scenarios (such as dealing with elderly patients, those unable to cough, or critically ill patients), completely eliminating unacceptable sputum is highly unrealistic. Therefore, ultimately, clinical lower respiratory tract pathogen diagnosis still inevitably relies on this vast volume of sputum samples, making a re-evaluation of the actual clinical value of unacceptable sputum particularly urgent.

In practice, when faced with specimen rejection, laboratories often rely solely on guidelines and consensus as explanations (Miller et al., 2024), lacking strong support based on large-scale real-world data. The specific impact of unacceptable sputum on culture positivity rates and microbiological results remains controversial: unacceptable specimens may lead to missed detection of true pathogens due to the dilution effect of oropharyngeal secretions or competition from other bacteria (Mandell et al., 2007); on the other hand, negative samples may produce “false positive” diagnostic noise due to the large-scale proliferation of oropharyngeal colonizing bacteria (Lee and Kim, 2020). Which of these two diametrically opposed patterns prevails in the real world? More importantly, is there a significant difference in the pathogen spectrum reported between acceptable and unacceptable sputum samples? Which specific pathogens are more likely to be detected in unacceptable specimens? And what exactly does improving the sputum sample qualification rate mean—which pathogens have seen an increase in their true detection rate?

To systematically answer these clinical challenges, this study retrospectively analyzed 143,101 sputum culture records with microscopic quality assessment records from our hospital over the past 10 years. Meanwhile, to more accurately define the diagnostic efficacy of sputum specimens, this study introduced data from bronchoalveolar lavage fluid (BALF), the sample most effective at excluding upper respiratory tract interference, as a benchmark (Hogea et al., 2020). This study aims to re-examine the true clinical significance of sputum specimen quality assessment through multi-dimensional big data comparison, providing solid evidence-based support for optimizing clinical microbiology testing strategies and guiding clinical decision-making.

## Materials and methods

### Study population and ethics

**FIGURE 1:**
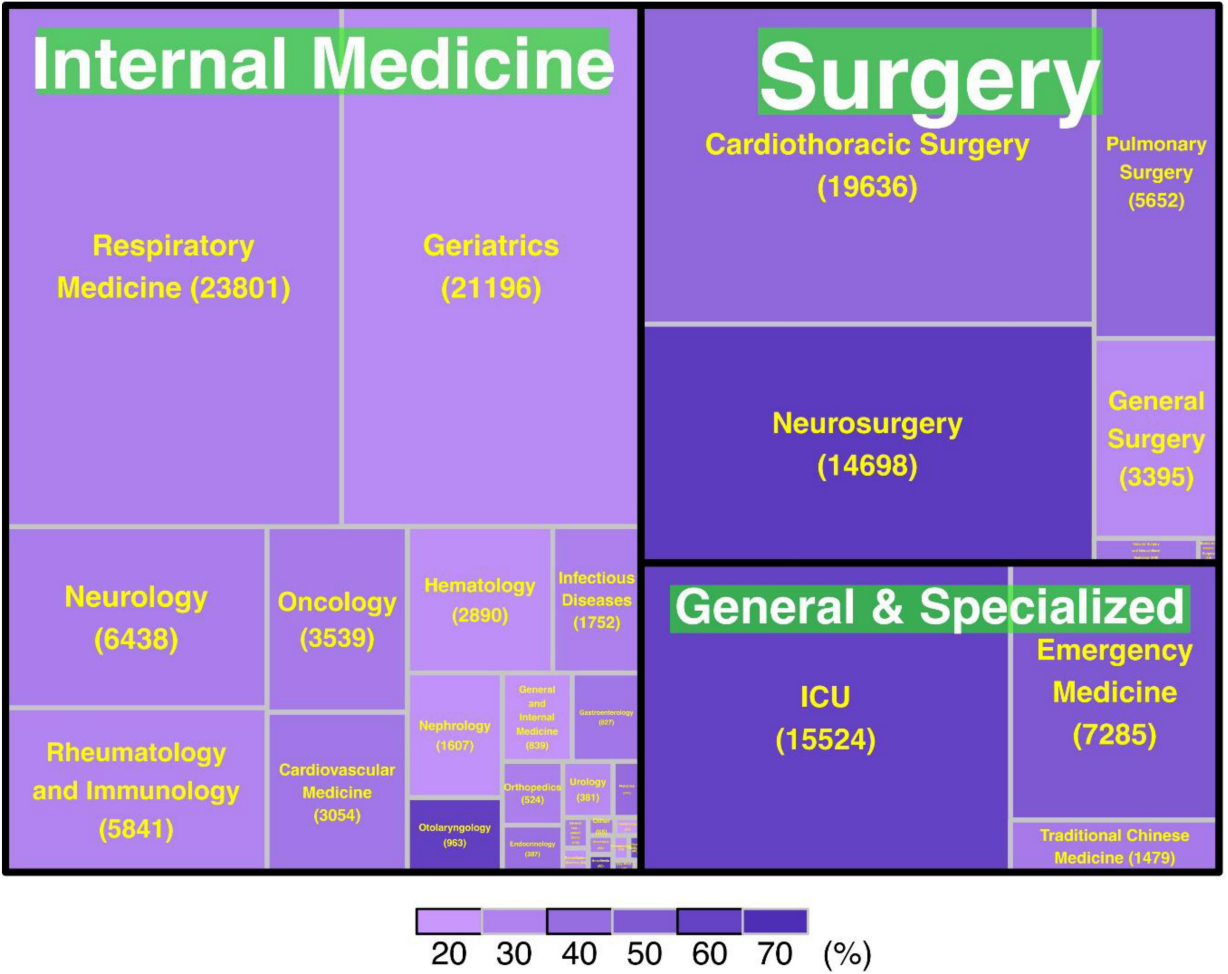
Treemap of department distribution and qualification rates of sputum samples. The area of each rectangle is proportional to the total number of submitted samples from the respective department (absolute sample sizes are indicated in parentheses). The intensity of the purple shade reflects the qualification rate, ranging from 20% to 70% as shown in the bottom color bar (darker purple represents higher qualification rates).

This retrospective study analyzed culture data from 143,101 sputum specimens with corresponding quality assessment records, collected between November 2015 and June 2025. Specimens were obtained from 35 clinical departments, including Respiratory Medicine, Geriatrics, Cardiothoracic Surgery, the Intensive Care Unit (ICU), Neurosurgery, and Emergency Medicine. The distribution and volume of specimens across these departments are illustrated in Figure 1. The study protocol was approved by the Institutional Review Board (IRB) of Nanjing Drum Tower Hospital, Nanjing, China (No. 2026-0015-01).

### Sputum quality assessment

To ensure diagnostic accuracy, sputum specimens were screened using the Murray-Washington grading system (Murray and Washington, 1975). The purulent portion of sputum was smeared, air-dried, and Gram-stained. Microscopic examination of stained smears was performed using a 40 × objective lens, observing 10 fields of view and calculating the average cell counts.

Acceptable sputum: Specimens containing > 25 leukocytes and < 10 squamous epithelial cells per low-power field (LPF) were validated as high-quality, representative samples.

Unacceptable sputum: Specimens containing > 10 squamous epithelial cells per LPF were categorized as having significant oropharyngeal contamination and deemed potentially contaminated, low-quality samples for definitive diagnostic interpretation.

### Sputum culture and identification

The sputum sample was simultaneously inoculated onto blood agar plates, chocolate agar plates, and MacConkey agar plates, followed by incubation a 37°C, 5% CO_2_ incubator. Microbial identification was performed using either the VITEK2-compact or VITEK^®^ MS system from bioMérieux France. Identification and antimicrobial susceptibility testing were completed using the VITEK2-compact fully automated microbial identification system from bioMérieux France. In accordance with the Clinical and Laboratory Standards Institute (CLSI) guidelines, *Methicillin-resistant Staphylococcus aureus* (MRSA) was inferred based on the cefoxitin screen test and oxacillin MICs, as determined and interpreted by the automated VITEK 2 system. Information on incubation duration, culture interpretation rules, reporting cutoffs, mixed-growth handling, the identification workflow (including VITEK/VITEK MS timelines), and AST breakpoints is available in the Supplementary materials.

### Data analysis

Raw sputum culture data were exported from the Laboratory Information System (LIS) into Excel format. Analysis was performed using R software (v4.4.1), with data read via the readxl package (v1.4.5). Data cleaning, organization, and summarization were conducted using the tidyverse package (v2.0.0). For visualization, Figure 1 (displaying classification proportions) was generated using the R treemap package (v2.4), while other statistical graphs were created with DataGraph software (v5.5). This study did not perform duplicate sample deduplication to preserve longitudinal dynamics in chronic infections and the true impact of sample quality during multiple hospitalizations on diagnosis.

## Results

### Departmental distribution and proportion of acceptable sputum

A total of 143,101 sputum culture results with sputum quality assessment records were collected from November 2015 to June 2025. The top five clinical departments by specimen volume were: the Department of Respiratory Medicine (*n* = 23801), Geriatrics (*n* = 21196), Cardiothoracic Surgery (*n* = 19636), the Intensive Care Unit (ICU, *n* = 15524), and Neurosurgery (*n* = 14698). The Department of Emergency Medicine followed as the sixth most frequent source (*n* = 7825; Figure 1).

Overall, acceptable sputum specimens accounted for 40.33% (*n* = 57718) of the total cohort, while the remainder were categorized as unacceptable sputum due to salivary contamination. When stratified by department, the highest proportions of acceptable sputum were observed in the Department of Neurosurgery (63.84%), the ICU (60.41%), and the Department of Emergency Medicine (50.45%). Additionally, although characterized by lower total sample volumes, the Department of Otolaryngology (*n* = 963) and the Department of Anesthesia (*n* = 60) also demonstrated high specimen quality rates (61.68% and 66.67%, respectively).

### Analysis of the relationship between sputum sample quality and culture-positivity rate

All specimens were stratified into two cohorts based on the Murray-Washington criteria. Statistical analysis demonstrated that the overall culture-positivity rate for acceptable sputum (*n* = 57718) was 57.28% (33063/57718), whereas the rate for unacceptable sputum (*n* = 85383) was 50.87% (43439/85383). The positive detection rate of acceptable sputum samples was 6.41% higher than that of unacceptable samples, with a statistically significant effect size observed (Odds Ratio = 1.29, 95% CI: 1.27–1.32, *P* < 0.001). This suggests that, overall, acceptable sputum samples exhibit a higher positive rate than unacceptable ones.

**FIGURE 2:**
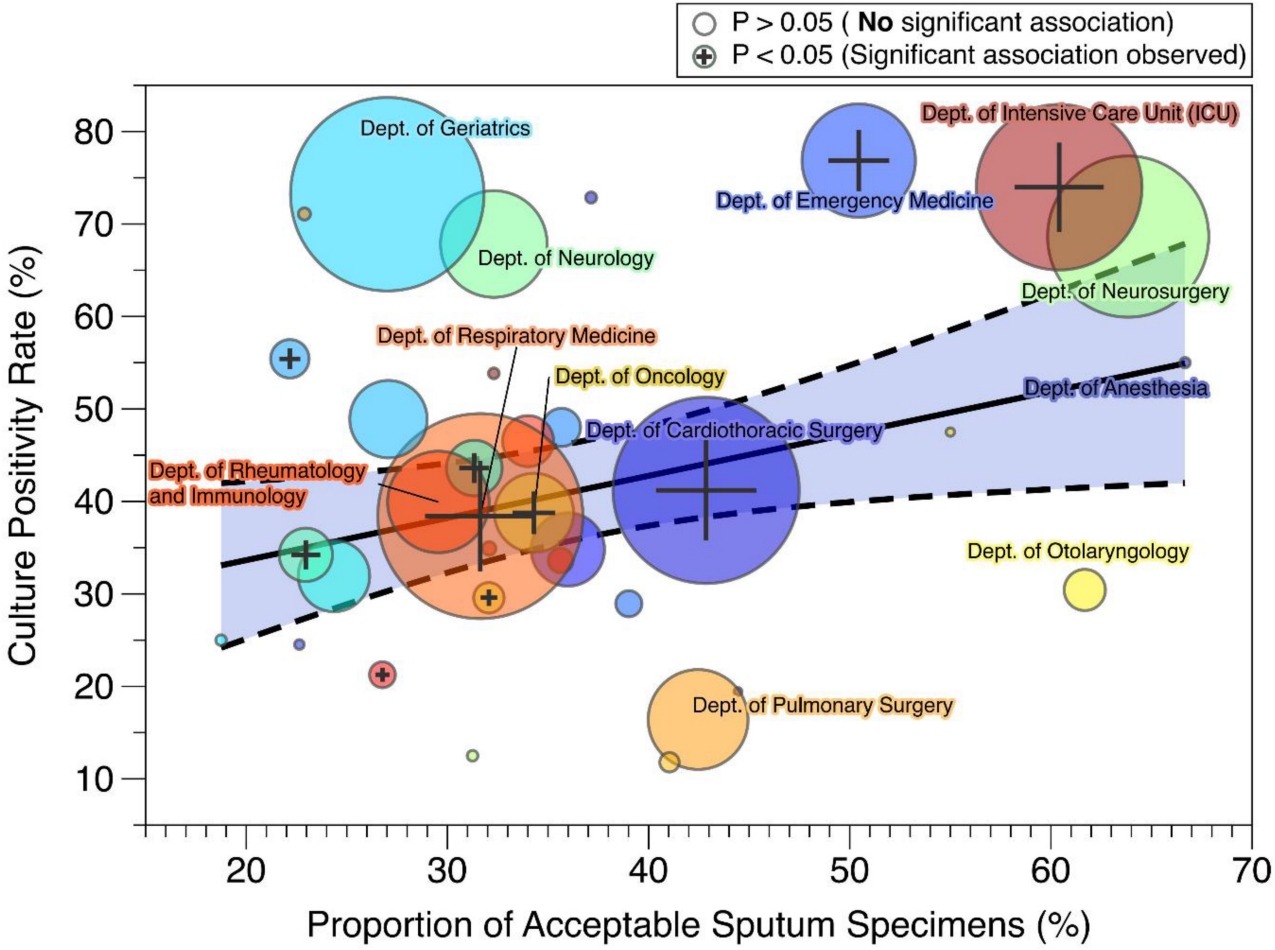
Distribution trend and linear regression analysis between sputum sample qualification rates and culture-positivity rates across clinical departments. Bubble size corresponds to the total volume of sputum specimens per department. A cross mark “+” in the center of scatters indicates a statistically significant difference in culture-positivity rate between acceptable and unacceptable samples (*P* < 0.05), while a plain circle indicates no significant difference (*P* > 0.05). The black solid line is the trend line showing a positive linear trend (trend line slope = 0.46), and the purple dashed lines are the 95% confidence interval of the trend line.

To determine whether this conclusion holds locally within each ward, all 35 wards in the hospital were treated as independent analysis units. The total sample volume, sputum qualification rate, and positive detection rate were calculated for each ward, and the correlation trend between these two factors was presented using a bubble scatter plot (Figure 2). Results showed that, at the department level, a positive linear trend was observed between sputum sample qualification rates and culture-positivity rates across various wards (trend line slope = 0.46). That is, higher ward-level qualification rates tended to correlate with higher culture-positivity rates, consistent with the overall sample analysis conclusion.

However, examining individual wards revealed that not all wards adhered to the principle of “higher qualification rate equating to higher positivity rate.” Among the 35 wards, only 10 demonstrated a statistically significant difference (*P* < 0.05) between the positivity rates of acceptable and unacceptable sputum samples. As shown in Figure 2, this observed difference was not directly correlated with the total sample volume, average positive detection rate, or overall sputum qualification rate of the corresponding wards. This indicates that although there is an overall positive correlation between “qualification rate and detection rate”, in specific wards, the quality of sputum samples does not consistently affect the positive detection rate, and no clear distribution pattern has been observed yet.

### Analysis of pathogen spectrum in sputum samples

Building upon the established fact that acceptable sputum exhibits higher culture-positivity rates, this study further analyzed differences in pathogen spectrum between acceptable and unacceptable sputum samples. The core question addressed was whether certain pathogens tend to appear in unacceptable sputum, potentially indicating upper respiratory tract contamination, or whether other pathogens were difficult to detect in unacceptable sputum, suggesting a higher likelihood of lower respiratory tract infection. To this end, the study incorporated alveolar lavage fluid culture results as a comparative benchmark.

### Dominant pathogens: higher detection rates in acceptable sputum samples

The top 10 bacteria by detection rate were: Klebsiella pneumoniae, Acinetobacter baumannii, Pseudomonas aeruginosa, Staphylococcus aureus, Stenotrophomonas maltophilia, Escherichia coli, Serratia marcescens, Proteus mirabilis, Enterobacter cloacae, and Klebsiella aerogenes.

All predominant pathogens exhibited consistent sample quality-related trends: acceptable sputum samples showed significantly higher detection rates than unacceptable sputum. Taking *Klebsiella pneumoniae*, the bacteria with the highest detection rate, as an example, its detection rate in acceptable sputum was 8.83% (5098/57718), while in unacceptable sputum it was 7.18% (6130/85383); *Acinetobacter baumannii* detection rate was 10.19% (5884/57718) in acceptable sputum and 6.21% (5305/85383) in unacceptable sputum; *Pseudomonas aeruginosa* detection rate in acceptable sputum: 8.13% (4691/57718); detection rate in unacceptable sputum: 6.91% (5899/85383).

### Microorganisms of special clinical significance: higher detection rates in acceptable sputum samples

**FIGURE 3:**
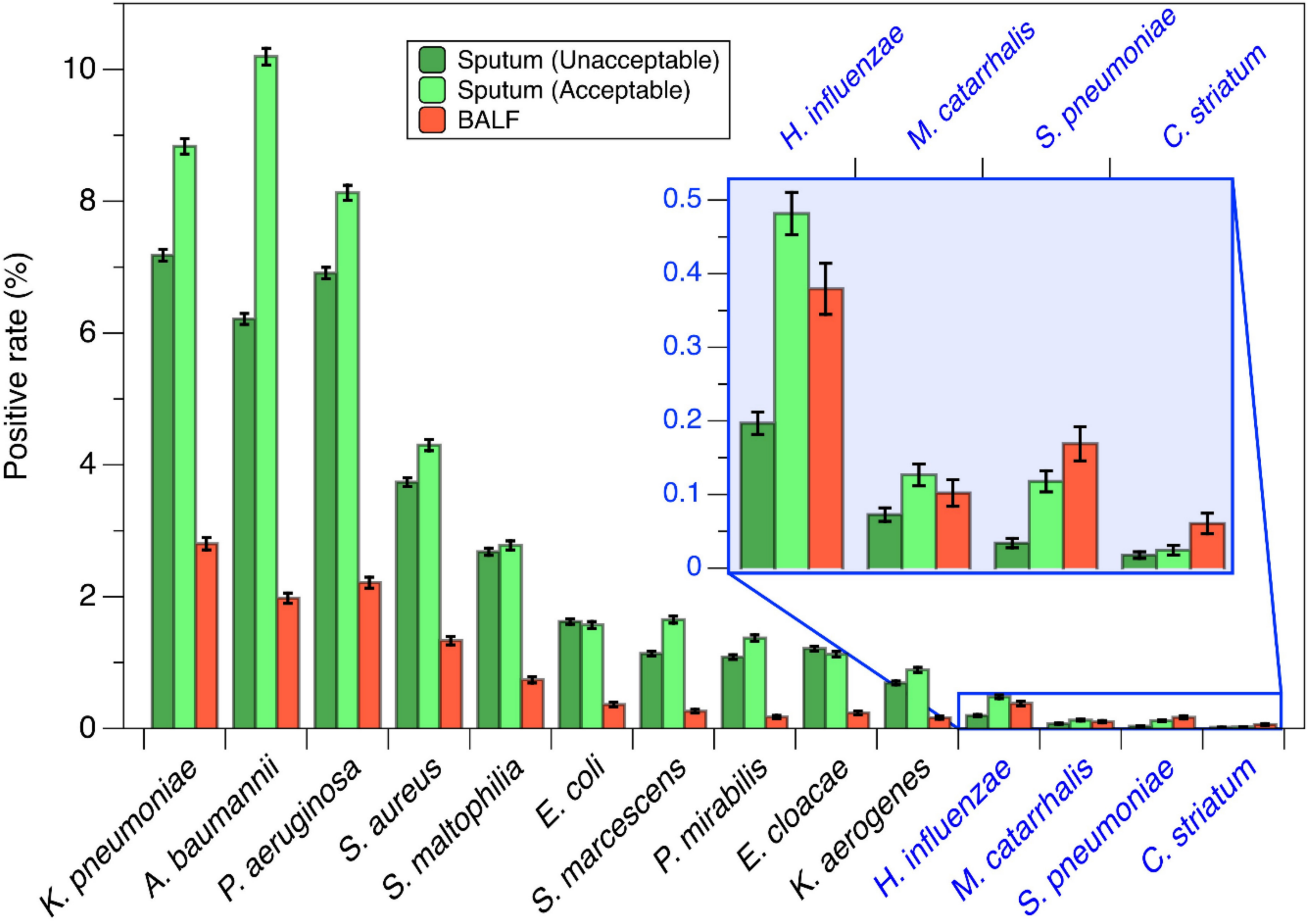
Comparison of bacterial detection rates in acceptable sputum, unacceptable sputum, and BALF. The figure compares the detection rates of major pathogens in acceptable sputum, unacceptable sputum, and BALF samples. Results indicate higher detection rates in acceptable sputum compared to unacceptable sputum. The inset graph shows detection trends for pathogens with low overall detection rates but high clinical significance (e.g., *H. influenzae, S. pneumoniae, M. catarrhalis, C. striatum*): BALF exhibits significantly higher detection rates.

Analysis of pathogens such as *Haemophilus influenzae, Streptococcus pneumoniae, Moraxella catarrhalis*, and *Corynebacterium striatum*—pathogens with “low overall detection rates but high clinical diagnostic value”—showed detection trends consistent with dominant pathogens (higher detection rates in acceptable sputum than in unacceptable sputum) and demonstrated superior detection efficacy in BALF (Figure 3).

### Culture-positivity characteristics: opportunistic pathogens significantly elevated in unacceptable sputum

Further analysis of distribution differences across all detected bacteria revealed a universal trend of “higher detection rates in acceptable sputum than unacceptable sputum.” No pathogen exhibited significantly higher detection rates in unacceptable sputum compared to acceptable sputum. Even for common oral/upper respiratory tract colonizers like *Staphylococcus epidermidis*, its detection rate in unacceptable sputum (8.12%) was only marginally higher than in acceptable sputum (5.35%). However, since these are often clinically classified as contaminating bacteria, they were excluded from pathogenic bacteria statistics. Moreover, the detection rate of such colonizers in BALF was extremely low (0.89%), further corroborating the characteristic that “unacceptable sputum is prone to upper respiratory tract contamination.

### Analysis of isolation rates of yeast-like fungi and aspergillus

In this study, fungal detection in respiratory specimens primarily involved yeast-like fungi and *Aspergillus* species. The detection rates for these two categories showed significant differences in association with sample quality and sample type.

### Detection of yeast-like fungi

**FIGURE 4:**
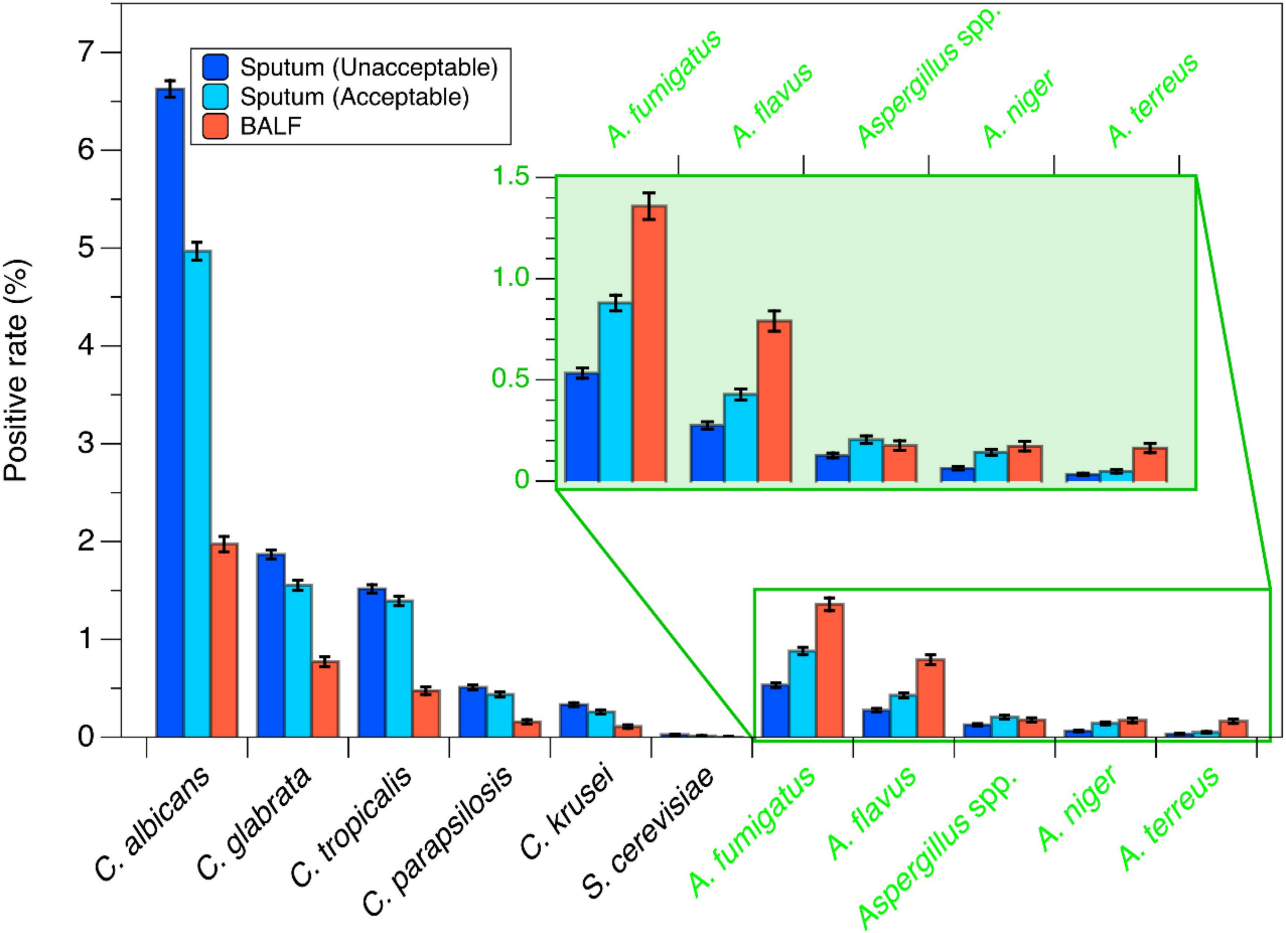
Analysis of the detection rate of fungi in acceptable sputum, unacceptable sputum, and BALF. Yeast-like fungi were primarily detected in sputum samples, with a significantly higher positive rate (9.97%) in sputum compared to BALF (3.49%). In contrast, the detection rate of *Aspergillus* species showed a decreasing trend: BALF (2.66%) > acceptable sputum (1.70%) > unacceptable sputum (1.03%).

A total of 143,101 sputum samples and corresponding BALF specimens were screened for yeast-like fungi, predominantly *Candida albicans, Candida glabrata, and Candida tropicalis*. Results indicate that all yeast-like fungi exhibit a consistent trend characterized by significantly higher isolation rates in unacceptable sputum compared to acceptable sputum. Taking *Candida albicans*—the most frequently detected species—as an example, its detection rate in unacceptable sputum was 6.63% (5658/85383), while in acceptable sputum it was 4.97% (2867/57718). Overall, the aggregate positivity rate for yeast-like fungi in sputum specimens (9.97%,14264/143,101) was markedly higher than that observed in BALF specimens (3.49%, 1094/31364) (Figure 4).

### Detection of aspergillus species

In contrast to yeast-like fungi, the positive detection rate for Aspergillus species showed a progressive decline trend: BALF > acceptable sputum > unacceptable sputum. In BALF samples, the detection rate of *Aspergillus* (primarily *Aspergillus fumigatus* and *Aspergillus flavus*) was 2.66% (834/31364). In acceptable sputum samples, the detection rate was 1.70% (983/57718), while in unacceptable sputum samples, it was only 1.03% (880/85383).

### Detection of rare fungi

Analysis of other rare fungi (e.g., *Cryptococcus, Mucor*) revealed a similar sample quality-related trend to *Aspergillus* species. However, due to the overall low detection rate (32 cases total), no statistically significant differences emerged. These findings are therefore excluded from the primary conclusions at this time.

### Analysis of key antibiotic resistance rates: differences in pathogenic bacterial resistance between sputum samples of varying quality and BALF

After establishing the impact of sputum sample quality on culture-positivity rates, we further analyzed differences in resistance rates to clinically critical antibiotics (imipenem, oxacillin) among pathogens detected in sputum samples of varying quality (acceptable/unacceptable sputum) and BALF (Table 1).

**TABLE 1:** Resistance rates of major pathogens in sputum and BALF specimens.

Bacterial species	Antibiotics	Total isolates	Unacceptable sputum	Acceptable sputum	BALF
*K. pneumoniae*	Imipenem	12077	1702/6116(27.83%)	1564/5082(30.78%)	131/879(14.90%)
*A. baumannii*	Imipenem	11711	3756/5264(71.35%)	4842/5832(83.02%)	524/615(85.20%)
*P. aeruginosa*	Imipenem	11055	2360/5807(40.64%)	1899/4565(41.60%)	199/683(29.14%)
*E. coli*	Imipenem	2407	64/1386(4.62%)	33/907(3.64%)	0/114(0.00%)
*S. marcescens*	Imipenem	1986	235/963(24.40%)	311/942(33.01%)	8/81(9.88%)
*E. cloacae*	Imipenem	1760	59/1036(5.69%)	25/650(3.85%)	6/74(8.11%)
*K. aerogenes*	Imipenem	1140	7/586(1.19%)	19/503(3.78%)	5/51(9.80%)
*P. mirabilis*	Imipenem	623	7/269(2.60%)	12/352(3.41%)	0/2(0.00%)
*K. oxytoca*	Imipenem	468	12/259(4.63%)	5/177(2.82%)	4/32(12.50%)
*C. koseri*	Imipenem	267	0/157(0.00%)	2/99(2.02%)	2/11(18.18%)
*S. aureus*	Oxacillin	6072	2269/3180(71.35%)	1717/2474(69.40%)	163/418(39.00%)

For the majority of Gram-negative bacilli, including *Klebsiella pneumoniae, Pseudomonas aeruginosa, Escherichia coli*, and *Serratia marcescens*, a consistent trend was observed: resistance rates were significantly lower in BALF compared to both sputum categories. Only in the comparison between acceptable and unacceptable sputum samples did *Klebsiella pneumoniae* and *Serratia marcescens* show a slight difference, with the acceptable sputum exhibiting a marginally higher resistance rate than the unacceptable sputum. The remaining strains (such as *Pseudomonas aeruginosa* and *Escherichia coli*) exhibited similar resistance rates in both sputum types. *Acinetobacter baumannii, Enterobacter cloacae*, and *Klebsiella pneumoniae* exhibit distinct patterns: *Acinetobacter baumannii* demonstrates a high overall resistance rate, with BALF resistance > acceptable resistance > unacceptable resistance; *Escherichia coli* and *Klebsiella pneumoniae* also exhibited higher BALF resistance rates than sputum samples. However, these strains were detected less frequently overall, necessitating further validation of this trend in clinical practice.

Among collected samples, *Staphylococcus aureus* exhibited consistently high resistance rates to oxacillin, with minimal difference between unacceptable and acceptable sputum samples. In BALF samples, the resistance rate of this bacterium decreased significantly, showing a reduction exceeding 30 percentage points compared to sputum samples. This indicates that the proportion of MRSA among S. aureus detected in BALF is substantially lower than that in sputum samples.

## Discussion

Despite the rapid evolution of molecular diagnostic technologies for respiratory infections, culture-based microbiological examination remains an irreplaceable cornerstone in global clinical practice (Lagier et al., 2015; Budayanti et al., 2019). Microbiology laboratories have long adhered to rigorous quality assessment standards based on the Murray-Washington Classification System, which utilizes the counts of squamous epithelial cells to polymorphonuclear leukocytes. However, when faced with the rejection of unacceptable specimens in clinical practice, physicians often harbor doubts: What exactly does an unacceptable sputum sample signify? Is it truly devoid of value? This study aims to systematically address this clinical challenge through a large-scale analysis by analyzing 143,101 sputum samples collected at Nanjing Gulou Hospital between 2015 and 2025, alongside concurrent BALF data.

First, this study addresses the clinical paradox raised in the introduction through large-scale data: Does unacceptable sputum cause a “false elevation” (colonization contamination) or a “false reduction” (dilution-induced missed detection) in positive rates? Results clearly show that the overall culture-positivity rate in acceptable sputum (57.28%) is significantly higher than in unacceptable sputum (50.87%). This data suggests a plausible interpretation that a “dilution and masking effect” potentially plays a significant role in unacceptable specimens. The substantial saliva contamination in unacceptable sputum may not only physically dilute the true pathogen load from the lower respiratory tract but, as a possible mechanism, also allow the accompanying oropharyngeal commensal flora to rapidly establish competitive growth dominance on culture plates (Nicholas and Djukanović, 2009). This overgrowth of extraneous flora creates substantial “diagnostic noise,” completely obscuring slower-growing or initially scarce true pathogens—particularly fastidious and characteristic pathogens like *Streptococcus pneumoniae* and *Haemophilus influenzae* (Jorth et al., 2019; Lu et al., 2020; Taylor et al., 2025). However, because this study did not directly measure organism load or plate overgrowth mechanisms, further prospective studies are warranted to confirm this hypothesis. Therefore, we contend that the greatest clinical harm of substandard specimens lies not only in misleading interpretations of colonizing bacteria but also in causing severe “missed diagnoses” of true pathogens.

Further analysis of the pathogen spectrum reveals significant “differentiation” in detection sensitivity across different pathogens based on specimen quality. Dominant pathogens such as Klebsiella pneumoniae, Acinetobacter baumannii, and Pseudomonas aeruginosa showed significantly higher detection rates in acceptable sputum samples. In contrast, the high detection of yeast-like fungi like *Candida albicans* in unacceptable sputum clearly confirms upper respiratory tract contamination. Notably, the distribution trend of *Aspergillus* species follows: BALF > compliant sputum > unacceptable sputum. This reveals a critical “diagnostic masking effect”: pathogens like *Aspergillus*, which have higher growth requirements or relatively low load, are easily obscured by background noise in unacceptable sputum. Based on these findings, this study concludes that while positive results in unacceptable sputum samples hold some clinical reference value, negative results cannot reliably rule out lower respiratory tract fungal or rare pathogenic bacterial infections.

Regarding the guideline recommendation to reject unacceptable sputum specimens, our study data offers a different perspective (Miller et al., 2024). Analysis reveals that even unacceptable samples maintain a culture-positivity rate exceeding 50%. Routine rejection of all unacceptable specimens would result in the loss of microbiological evidence for some patients, potentially leading to a deficit in diagnostic information. In many scenarios—such as with elderly patients, those with impaired consciousness, or individuals unable to undergo invasive procedures—an unacceptable sputum sample often represents the sole diagnostic clue. The loss of diagnostic information due to sample rejection presents a significant clinical challenge. Research indicates that sputum culture, regardless of sample qualification, exhibits similar specificity across all specimens, enhancing the diagnostic value for community-acquired pneumococcal pneumonia in the elderly (Budayanti et al., 2019). Therefore, we contend that unacceptable sputum is not “unusable” but requires “differentiated interpretation” considering specimen quality context.

Regarding antimicrobial stewardship, antimicrobial susceptibility results from specimens of varying quality show significant divergence (Grabe and Resman, 2019). For *Klebsiella pneumoniae, Pseudomonas aeruginosa*, and MRSA, resistance rates detected in sputum are significantly higher than in BALF. This indicates sputum culture results are highly susceptible to interference from highly resistant bacteria colonizing the upper respiratory tract. Consequently, they significantly overestimate the actual antimicrobial resistance risk in lower respiratory tract infections, potentially leading to unnecessary clinical selection of higher-tier antimicrobial agents. However, for Acinetobacter baumannii, BALF exhibited the highest resistance rate. These findings underscore a dual responsibility: laboratory departments should provide interpretive reports considering sample quality context, while clinicians must exercise caution when interpreting antimicrobial susceptibility reports from specimens of varying quality. Individualized interpretation based on pathogen type is essential to avoid blind escalation of antimicrobial therapy.

In the pathogenetic diagnosis of fungi, this study reveals an even more pronounced “sample quality dependency” than that observed in bacteria, providing exceptionally clear big data support for clinically distinguishing fungal “colonization” from “infection.” The detection rate of yeast-like fungi, represented by *Candida albicans*, is significantly higher in substandard sputum samples than in acceptable sputum and BALF samples. This finding is highly consistent with their biological characteristics as common commensal colonizers of the oropharynx. This data strongly corroborates that yeast-like fungi cultured from unacceptable sputum samples most likely originate from upper respiratory tract contamination. Clinicians should exercise extreme caution when encountering such positive reports and avoid initiating antifungal therapy indiscriminately.

However, filamentous fungi represented by *Aspergillus* exhibit a completely opposite detection gradient (BALF > compliant sputum > unacceptable sputum). *Aspergillus* often invades lung tissues and grows slowly on conventional culture media, making it highly susceptible to being engulfed or masked by background contaminants in unacceptable specimens (Pérez-Cantero et al., 2020). Given this pronounced differentiation trend, this study strongly recommends that when invasive pulmonary aspergillosis or other invasive fungal infections are clinically suspected, one must never rely on “negative culture results” from substandard sputum specimens to rule out the diagnosis. In such cases, actively obtaining high-quality, acceptable sputum samples or promptly performing bronchoscopy to collect BALF is the core strategy to prevent missed diagnoses of invasive fungal infections. The patterns of fungal detection exhibit high intrinsic consistency with bacterial detection: poorer sample quality tends to expose oropharyngeal colonizing flora, while higher-quality samples better filter out background noise to identify true lower respiratory tract pathogens.

Several limitations of this study should be considered when interpreting the findings. First, as a single-center retrospective analysis, the results reflect the clinical practices and patient population of one institution and may not be directly generalizable to settings with different laboratory protocols or case mixes. Nevertheless, the inclusion of 35 clinical departments across a 10-year period provides substantial internal diversity. Second, routine culture-based methods do not detect fastidious organisms requiring specialized media or non-culture techniques — most notably Mycobacterium tuberculosis, Pneumocystis jirovecii, and Legionella species — and therefore this study cannot address the relationship between sputum quality and the detection of these pathogens. Third, in the absence of clinical adjudication, culture results were classified by microbiological criteria alone, without distinguishing infection from colonization or contamination; the observed differences in yeast-like fungi and Aspergillus detection across specimen types are consistent with this inherent limitation and should be interpreted with that context in mind. Fourth, patient-level deduplication was intentionally not performed, as repeated specimens from the same patient during multiple hospitalizations capture clinically meaningful longitudinal dynamics; however, this approach may cause certain patient subgroups to be overrepresented in aggregate statistics. Fifth, the sputum-BALF comparison was not derived from matched pairs, which limits direct intra-subject comparisons but preserves the real-world distribution of specimen types and a broader patient coverage. Finally, the 10-year study span encompasses inevitable evolution in laboratory workflows, personnel expertise, and antimicrobial susceptibility breakpoints, all of which may introduce temporal heterogeneity into the data. Additionally, due to historical data constraints, the evaluation of microscopic phagocytosis patterns was not included in our specimen quality assessment. Despite these limitations, the large sample size (143,101 sputum specimens with quality assessment records), the decade-long observation window, and the multi-dimensional analytic framework — encompassing culture-positivity rates, species-level spectrum differences, and antimicrobial resistance patterns — provide robust descriptive evidence that can inform cautious, context-aware interpretation of sputum culture results across the quality spectrum, particularly for patients from whom better specimens cannot feasibly be obtained.

This study re-examines the classic paradigm of sputum specimen quality assessment through a robust, decade-long retrospective analysis of large-scale clinical microbiology data. Our findings demonstrate that while strict adherence to quality screening standards is paramount for diagnostic accuracy, mechanically rejecting all unacceptable specimens may inadvertently deprive patients with sample difficulties—such as the elderly or critically ill—of vital microbiological clues. Crucially, this work is the first to uncover systematic variations in antimicrobial susceptibility outcomes stratified by specimen quality at a big-data scale, offering a novel research trajectory for optimizing future antimicrobial stewardship and laboratory interpretive reporting.

## Data Availability

The datasets presented in this article are not readily available because the data sets used and analyzed during the current study are available from the corresponding author upon reasonable request. Requests to access the datasets should be directed to liuchangbio@163.com.
