# Supplementary material for: The impact of sputum sample quality on sputum culture results: a retrospective analysis of clinical microbiology sputum culture data spanning 10 years
Source: Front Microbiol. 2026 Jul 7;17:1894814. doi: 10.3389/fmicb.2026.1894814 (PMC13385188; doi:10.3389/fmicb.2026.1894814)
Supplement: Supplementary file 1 [file Presentation_1.pdf]

Supplementary material

Table 1. Distribution of unique patients and frequency of repeat sputum and BALF sampling over the 10-year study period

Number of sample repetitions	Number of samplings per patient (*n*)	Percentage (%)	Cumulative Percentage (%)
1	31,058	59.0%	59.0%
2	12,941	24.6%	83.6%
3	2,797	5.3%	88.9%
4-5	3,091	5.9%	94.8%
6-10	1,880	3.6%	98.4%
11-20	612	1.2%	99.6%
21-50	207	0.3%	99.9%
>50	59	0.1%	100.0%

Detailed Microbiological and Susceptibility Methodologies

1. Specimen Processing and Incubation Duration

Upon arrival at the clinical microbiology laboratory. To ensure diagnostic accuracy, sputum specimens were screened using the Murray-Washington grading system. The purulent portion of sputum was smeared, air-dried, and Gram-stained. Microscopic examination of stained smears was performed using a 40× objective lens, observing 10 fields of view and calculating the average cell counts. The sputum sample was simultaneously

inoculated onto blood agar plates, chocolate agar plates, and MacConkey agar plates, followed by incubation in a 37°C, 5% CO_2 incubator.

Incubation Duration: Routine cultures were inspected daily after a minimum of 18 hours of incubation. In the absence of significant bacterial growth, the incubation was prolonged. A final report of 'no bacterial growth after two days of incubation' was issued if no growth occurred after at least 48 hours. Upon detection of bacterial growth, identification and antimicrobial susceptibility testing (AST) were performed directly for pure cultures; mixed cultures were first subcultured to achieve isolation prior to subsequent identification and testing.

2. Culture Interpretation Rules and Reporting Thresholds

The interpretation of sputum cultures was strictly governed by standardized laboratory operating procedures designed to differentiate true lower respiratory tract pathogens from upper-airway commensal flora:

- Quantitative Thresholds: For routine sputum specimens, bacterial growth was quantified quantitatively (+, ++, +++).Pathogen identification and antimicrobial susceptibility testing (AST) were triggered only when a potential pathogen was recovered in moderate-to-abundant growth.
- Commensal Isolation: The isolation of typical upper respiratory tract microbiota (e.g., alpha-hemolytic streptococci, *Neisseria* spp.,

Corynebacterium spp.) in low quantities without matching microscopic leukocytosis was interpreted as normal oral flora contamination and was not reported with AST.

3. Handling of Mixed Growth

Sputum samples displaying polymicrobial growth were evaluated cautiously to prevent the reporting of clinically irrelevant contaminants:

- Two or Fewer Potential Pathogens: If two distinct potential pathogens (e.g., *Klebsiella pneumoniae* and *Pseudomonas aeruginosa*) were isolated, and both met the reporting threshold, both organisms were fully identified, and AST was performed for each.
- Three or More Organisms (Mixed Growth): Plates exhibiting growth of three or more different morphological types of Gram-negative bacilli or potential pathogens were classified as "mixed upper respiratory flora/contaminated growth." These were not subjected to full identification or AST, and a request for a repeat specimen was issued, unless one specific morphotype was overwhelmingly predominant.

4. Pathogen Identification Workflow & VITEK/VITEK MS Timeline

Over the 10-year study period (2015–2025), the laboratory identification workflow evolved alongside advancements in diagnostic technology:

- Phase 1 (2015 – 2019): Microbial identification was primarily driven by biochemical profiling utilizing the VITEK 2 Compact automated system (bioMérieux, Marcy-l'Étoile, France) with ID-GNB (Gram-negative bacilli) and ID-GP (Gram-positive cocci) cards. Supplementary manual phenotypic tests (e.g., catalase, oxidase, coagulase) were used for confirmation.
- Phase 2 (2020 – 2025): The laboratory transitioned to Matrix-Assisted Laser Desorption Ionization–Time of Flight Mass Spectrometry (MALDI-TOF MS) utilizing the VITEK MS system (bioMérieux, France) as the primary first-line identification tool. The VITEK 2 system was retained for automated AST and back-up identification for rare or challenging isolates. The workflow transition was fully validated internally to ensure total longitudinal consistency in genus- and species-level naming.

5. AST Breakpoint Standards and Historical Data Integration

- AST Platforms: Antimicrobial susceptibility testing was performed using the automated VITEK 2 system (AST-GN and AST-GP cards). Disk diffusion (Kirby-Bauer) or Etest methods were employed as supplemental or confirmatory methods for specific drug-bug combinations or when automated results required verification.

- Breakpoint Standards: Minimum Inhibitory Concentrations (MICs) and zone diameters were interpreted strictly according to the Clinical and Laboratory Standards Institute (CLSI) performance standards relevant to each testing year.
- Historical Data Handling: Because this study is a long-term retrospective registry analysis, historical AST interpretations (Susceptible, Intermediate, Resistant) were extracted directly from the hospital laboratory information system (LIS) as originally classified at the time of patient care. Results were not retrospectively reinterpreted using current 2026 breakpoints. This approach preserves the historical epidemiological realities of contemporary clinical decision-making.
